# Knowledge and Perceptions about Nicotine, Nicotine Replacement Therapies and Electronic Cigarettes among Healthcare Professionals in Greece

**DOI:** 10.3390/ijerph13050514

**Published:** 2016-05-20

**Authors:** Anastasia Moysidou, Konstantinos E. Farsalinos, Vassilis Voudris, Kyriakoula Merakou, Kallirrhoe Kourea, Anastasia Barbouni

**Affiliations:** 1 National School of Public Health, Alexandras Av. 196, Athens 11521, Greece; ste.mois@hotmail.com (A.M.); kmerakou@esdy.edu.gr (K.M.); kallirrhoe_kourea@yahoo.gr (K.K.); abarbouni@esdy.edu.gr (A.B.); 2 Onassis Cardiac Surgery Center, Sygrou 356, Kallithea 17674, Greece; vvoudris@otenet.gr

**Keywords:** smoking/harm reduction, nicotine, electronic cigarettes, healthcare, physicians, nurses

## Abstract

*Introduction*. The purpose of this study was to evaluate the knowledge and perceptions of Greek healthcare professionals about nicotine, nicotine replacement therapies and electronic cigarettes. *Methods*. An online survey was performed, in which physicians and nurses working in private and public healthcare sectors in Athens-Greece were asked to participate through email invitations. A knowledge score was calculated by scoring the correct answers to specific questions with 1 point. *Results*. A total of 262 healthcare professionals were included to the analysis. Most had daily contact with smokers in their working environment. About half of them considered that nicotine has an extremely or very important contribution to smoking-related disease. More than 30% considered nicotine replacement therapies equally or more addictive than smoking, 76.7% overestimated their smoking cessation efficacy and only 21.0% would recommend them as long-term smoking substitutes. For electronic cigarettes, 45.0% considered them equally or more addictive than smoking and 24.4% equally or more harmful than tobacco cigarettes. Additionally, 35.5% thought they involve combustion while the majority responded that nicotine in electronic cigarettes is synthetically produced. Only 14.5% knew about the pending European regulation, but 33.2% have recommended them to smokers in the past. Still, more than 40% would not recommend electronic cigarettes to smokers unwilling or unable to quit smoking with currently approved medications. Cardiologists and respiratory physicians, who are responsible for smoking cessation therapy in Greece, were even more reluctant to recommend electronic cigarettes to this subpopulation of smokers compared to all other participants. The knowledge score of the whole study sample was 7.7 (SD: 2.4) out of a maximum score of 16. Higher score was associated with specific physician specialties. *Conclusions*. Greek healthcare professionals appear to overestimate the adverse effects of nicotine, and many would not recommend any nicotine-containing product as a long-term smoking substitute. Additionally, they have poor knowledge about the function and characteristics of electronic cigarettes.

## 1. Introduction

Smoking is an important reversible cause of morbidity and mortality in the modern world, with the World Health Organization (WHO) expecting one billion smoking-related deaths in the 21st century. Smoking is a serious public health threat, even among developed European countries [1,2] where intensive tobacco control measures have been implemented in recent years. The currently available smoking cessation medications have limited success. Nicotine substitutes have smoking cessation rates below 7% at 1 year [3,4], although combination therapy with multiple nicotine replacement products may be more effective [5]. Oral medications have less than 20% success rate in well-organized clinical studies [6], and are usually less effective in everyday clinical practice [7]. As a result, smoking prevalence remains high, with the latest data estimating that, in 2014, 26% of the population >15 years in the European Union (EU) smokes [2], compared to 28% in 2012 [8].

The situation in Greece concerning smoking is particularly worrisome, with 38% of the population smoking in 2014 [2] compared to 40% in 2012 [8]. This is happening despite the intense efforts of the local tobacco control community and regulators, which has resulted in the implementation of public places smoke-free laws, warnings on tobacco cigarette packaging and media campaigns educating the population about the adverse effects of smoking. Additionally, a network of specialized smoking cessation clinics (in both public and private healthcare sectors) has been developed, which is mainly managed by respiratory physicians and, to a somewhat lesser extent, by cardiologists.

Tobacco harm-reduction, a strategy of providing less harmful alternative products to smokers to substitute the use of combustible tobacco, have been developed in an effort to accelerate the reduction in smoking prevalence and reduce smoking-related disease and death [9]. This is based on the fact that nicotine, although contributing to dependence, is of low health-harm potential when obtained through a non-combustible product. According to the International Agency for Research on Cancer (IARC), nicotine is not classified as a carcinogen [10]. Several laboratory studies have identified a tumor-promoting effect of nicotine [11,12] as well as an effect on atherosclerosis [13,14]. However, clinical studies have failed to reproduce these findings. The Lung Health Study followed up a cohort of 3320 users of nicotine replacement therapy (nicotine gum) for 7.5 years and found that smoking, but not nicotine replacement therapy, was a predictor of lung cancer [15]. The same study group found that, after 5 years of follow-up, use of nicotine gum was not associated with higher rates of hospitalizations for cardiovascular conditions and cardiovascular deaths [16]. A recent metanalysis of the cardiovascular effects of smoking cessation pharmacotherapies evaluated 21 randomized controlled trials of nicotine replacement therapies and found an elevated risk of all cardiovascular events which was driven predominantly by less serious events, mainly tachycardia [17]. More extensive epidemiological data on the association between nicotine intake and cancer or cardiovascular disease are available from studying snus use. Snus users obtain equal or higher amounts of nicotine on a daily basis compared to smokers [18,19]. However, snus use, especially Scandinavian snus which is a low-nitrosamine smokeless tobacco product, carries a very low risk of developing cancer compared to smoking. Luo *et al.* found that the risk of oral and lung cancer among snus users was similar to never smokers, while only pancreatic cancer risk was elevated in current snus users and those using more than 10 grams per day [20]. A systematic review of the relation between snus use and cancer concluded that, if all smoking population was instead using snus, only 1.1% of the smoking-attributed cancers would occur [21], showing that the risk for cancer is higher than non-use but minimal compared to smoking. Similar evidence exists for the association between snus use and cardiovascular disease. A retrospective case control study in Sweden found that the risk for acute myocardial infarction was similar between snus users and never smokers [22]. An analysis of eight prospective studies also found no association between snus use and acute myocardial infarction [23]. Similarly, an analysis of eight prospective cohort studies on stroke risk (ischemic and hemorrhagic), with more than 130,000 non-smoking men participating, found no association between snus use and risk of stroke [24]. These studies have shown that nicotine is highly unlikely to contribute significantly to smoking-related cancer and cardiovascular disease.

Electronic cigarettes are the latest addition in tobacco harm reduction [25]. These battery devices produce an aerosol by heating a liquid which may contain nicotine and other food-approved ingredients, including flavors; the aerosol is subsequently inhaled by the user. Despite the lack of long-term epidemiological studies, currently-available evidence from chemistry, toxicology and few short-term clinical studies suggests that electronic cigarettes are by far less harmful than smoking [26,27]. This is mostly related to the lack of combustion and the low temperatures of evaporation, resulting in far lower toxic emissions compared to tobacco cigarette smoke [28,29,30]. The efficacy of electronic cigarettes on smoking cessation has been questioned. Two randomized controlled trials have shown a small effect of electronic cigarettes on smoking cessation [31,32]. However, both used outdated and ineffective products. Moreover, the design of randomized controlled trials, which dictates the use of a single product by all participants, may be inappropriate to evaluate the overall efficacy of electronic cigarettes on smoking cessation considering that switching from smoking to electronic cigarette use is related to a behavioral change and that there is a huge variability of electronic cigarette products which are selected by users based on self-preference. A recent study using more advanced products showed substantially higher cessation rate [33], while a population study in the UK showed that they are substantially more effective compared to nicotine replacement therapies [34].

The role of healthcare professionals in properly advising and guiding the smoking population has long been established. A meta-analysis of 31 studies which included over 26,000 smokers found that even brief advice increased the chances of smoking cessation [35]. Another meta-analysis concluded that counseling of smokers by nurses increases the chances of quitting by 29% [36]. A prerequisite for effective counseling to smokers is proper education and knowledge of healthcare professionals about the effects of smoking on health and about all the available methods and products that could contribute to smoking reduction or cessation. Healthcare professionals are the most important source of information about the relative risks of different products for smokers, due to the respect and corresponding confidence enjoyed by society. Current evidence shows that the knowledge and concepts of healthcare professionals about smoking and nicotine are not always appropriate and science-based. A study of physicians from UK and Sweden showed that a large proportion (40%) of respondents incorrectly believed that nicotine was the most important or second most important cause of smoking-related disease [37]. This can have negative effects on the management of smokers. For example, it could discourage smokers from using pharmaceutical nicotine preparations or other nicotine-containing products which are less harmful than smoking. Studies in Norway and in other countries showed that the population overestimates the harmful effects of smokeless tobacco (snus) [38,39,40] and nicotine replacement therapies [41] compared to smoking, and this seems to be related to misconceptions about the relative risks of different products by healthcare professionals [42].

Considering the exponential growth in awareness and use of electronic cigarettes over the past few years [43,44], it is expected that, over time, more smokers will seek advice from healthcare professionals about these alternative to smoking products. Therefore, the purpose of this study was to evaluate the concepts and beliefs of a convenient sample of Greek healthcare professionals about nicotine, nicotine replacement therapies and electronic cigarettes through an online questionnaire.

## 2. Methods

A questionnaire was developed by the researchers and was uploaded to an online survey tool (www.surveymonkey.com). Initially, participants had to read a brief presentation of the research purpose and agree to an informed consent. Then they were transferred to the questions of the survey. The questionnaire had five main sections, asking for information about: (1) demographics of the participants, including age, gender, profession, working time and working sector (public or private); (2) smoking status of the participants; (3) perceptions and knowledge about the contribution of nicotine to smoking-related disease; (4) knowledge about the efficacy and dependence potential of nicotine replacement therapies, and; (5) knowledge about electronic cigarettes. Concerning nicotine, the main purpose was to evaluate the perceived relative risk of nicotine exposure compared to exposure to other smoking-related toxins, and its contribution to smoking-related disease. For nicotine replacement therapies, questions were mainly focused on the willingness to propose the long-term use as smoking substitutes (as suggested by recent guidelines [45,46,47]), their dependence potential compared to smoking and the knowledge about their efficacy in smoking cessation. Since electronic cigarettes are novel products, our purpose was to evaluate the knowledge about their functional and design characteristics, nicotine source in e-liquids and pending EU regulation, and evaluate whether healthcare professionals would recommend them as a substitute for smoking in those unable or unwilling to quit with other methods. Finally, participants were asked to report what they would consider appropriate regulation for electronic cigarettes. The questionnaire of the survey is presented in Supplementary materials. To improve the questionnaire design and ensure the comprehension of the survey items, five healthcare professionals (two cardiologists, one respiratory physician, one general practitioner and one nurse) were recruited and the assessment of the questionnaire was performed using the method of cognitive interviewing [48]. This process occurred before the questionnaire was released online. The study was approved by the ethics committee of the National School of Public Health in Athens, Greece (Ethics Committee Approval Code: 1701/16-12-2014). The questionnaire was anonymous and participants were informed through the consent form that they could exit the questionnaire at any time. The IP addresses were recorded with the only purpose to remove double entries.

A personal invitation to participate, together with the link to the questionnaire, was sent by email to a convenient sample of physicians and nurses working at the seven largest (in terms of bed capacity) hospitals and eight primary care centers located in Athens, Greece. Additionally, private practice physicians and nurses were selected through online search tools. The purpose of the hospitals and primary care centers selection was to cover all major districts of Athens, while private practice physicians and nurses were selected from the same areas. In an effort to balance the selection process, a similar number of public and private sector professionals were invited from each Athens district. The selection was based on availability of email addresses, and no other communication tool (telephone, personal contact) was used for survey participation. A total of 865 healthcare professionals were contacted. For physicians, general practitioners, cardiologists, dentists, internists, pediatricians and respiratory physicians were asked to participate. The main reasons for recruiting cardiologists and respiratory physicians were that they treat the most common smoking-related diseases (cardiovascular and respiratory diseases) and that they are directing smoking cessation centers in Greece. Other specializations were also selected based on their interaction with smokers and smoking-related disease. The only exclusion criteria were not responding to the question about occupation (Supplementary materials, question 3) and responding to less than 80% of the questions.

### Statistical Analysis

Results were reported for the whole sample, with categorical variables presented as number (%) and continuous variables as mean (SD). Subsequently, comparisons were made between smokers (current and former) and never-smokers, between physicians and nurses and between a subgroup of physicians working in tobacco cessation in Greece (cardiologists and respiratory physicians) and all other participating healthcare professionals. The reason for dividing the sample into these subgroups is related to the expectation that smokers would have better knowledge about the study subject due to personal experience and use, physicians would be better educated about health issues related to smoking and cardiologists and respiratory physicians would also be better educated since they are responsible for treating the major causes of smoking-related morbidity and mortality and they manage the smoking cessation clinics in Greece. Comparisons between groups were performed with independent-samples *t*-test, one way ANOVA and χ^2^ test. A “knowledge score” was calculated for each participant, by scoring 1 point for every correct answer of the participants in some of the questions (see Supplementary materials). Univariate and multivariate linear regression analysis was performed to explore the association between the knowledge score and various characteristics of the study participants. All variables with *p* < 0.05 in univariate analysis were included in the multivariate model. A *p* value of <0.05 was considered statistically significant and all analyses were performed with commercially available software (SPSS v22.0, Chicago, IL, USA).

## 3. Results

### 3.1. Descriptive Analysis of the Study Sample

The results of the descriptive analysis of the whole study sample are shown in Table 1. From the 865 healthcare professionals invited, 316 entered the survey questionnaire. After excluding double entries (through the IP addresses), rejections to the informed consent, participants not responding to the question about profession and participants responding to less than 80% of the questions, a total of 262 subjects (77 were nurses and 185 were physicians) were included in the analysis (response rate of 30.3%). Healthcare professionals from all hospitals and primary care centers participated in the study. There was no statistical difference in gender, profession and working sector between the participants and those who were excluded or did not enter the survey. Most physicians were cardiologists, followed by respiratory physicians. More than half of the participants were working in the public healthcare sector, while the average working time was 12 years. The vast majority said they had daily contact with smoking patients in their working environment, while a high proportion considered they had very of fairly high level of knowledge about smoking. More than 50% were current or former smokers. Among former smokers, most had stopped smoking without the use of any aid, while seven participants reported smoking cessation with the use of electronic cigarettes.

Participants were asked to score the risk for several tobacco and alternative products, using a scale from 1 (minimum risk) to 10 (maximum risk). The following products were listed: tobacco cigarettes, snus, electronic cigarettes, nicotine replacement therapies and oral smoking cessation medications. Significant differences were found in risk scores among different products (*p* < 0.001). As expected, participants gave the highest risk score to tobacco cigarettes. The risk score for snus was very close to that of tobacco cigarettes while the risk score for electronic cigarettes was lower than both tobacco cigarettes and snus. Nicotine replacement therapies and oral smoking cessation medications had the lowest scores, with no statistically significant difference between them.

Participants were also asked to score the risk from several tobacco cigarette components, again using a scale from 1 (minimum risk) to 10 (maximum risk). The following components were listed: nicotine, inhaled smoke, carbon monoxide, tar and tobacco. Statistically significant differences were observed in risk scores among the different components (*p* < 0.001). The highest score was recorded for tar while slightly lower scores were given for smoke and carbon monoxide, without statistically significant difference between them. The risk score for nicotine was 8.0 (2.1) and was ranked 4th in terms of risk among the five components of smoking listed. Tobacco was considered by the participants as the component with the lowest risk.

More than half of the participants reported that nicotine has an extremely or very important contribution to the development of smoking-related diseases, mainly lung cancer and atherosclerosis, while approximately 45% considered extremely or very important the contribution of nicotine to the development of cancer to other than the lung organs. Almost four out of five participants considered nicotine replacement therapies less harmful than smoking; 6.5% said they did not know and 17.0% considered them equally or more harmful than smoking. About one out of three participants considered nicotine replacement therapies more addictive than smoking. Participants overestimated the efficacy of nicotine replacement therapies on smoking cessation, with just 23% correctly responding that their success rate is less than 10% at one year. The vast majority believed that nicotine in nicotine replacement therapies is synthetically-produced rather than extracted from the tobacco plant. Only one out of five participants would recommend the long-term (over 6 months) use of nicotine replacement therapies as an aid to reduce or quit smoking.

Concerning electronic cigarettes, one out of three participants reported that they have recommended the use of the electronic cigarettes to smokers in the past. The majority believed that electronic cigarettes are less harmful than smoking; only 1.5% of the participants considered them more harmful than smoking. Almost half, however, considered electronic cigarettes equally or more addictive than smoking. As with nicotine replacement therapies, most participants thought that the nicotine present in electronic cigarettes is synthetically-produced. One out of three believed that electronic cigarettes involve combustion, while the majority believed that the ingredients are approved for inhalational use. More than half reported they would recommend electronic cigarette use to smokers who have failed to stop smoking by other means, while about 55% would also recommend it to smokers who refuse to use pharmaceutical methods for smoking cessation. Ιt was noteworthy that less than 15% of the respondents knew the EU legislation on electronic cigarettes. The majority believed that electronic cigarettes sales to minors should be banned, while about 40% recommended to ban nicotine, ban advertising, be licensed as pharmaceutical products and include a warning on the label that they are equally harmful to smoking.

### 3.2. Comparison between Smoking and Never Smoking Participants

The results of the comparison between smoking (current and former) and never smoking participants are presented in Table 2. There was no difference between groups in the number of physicians and nurses, working time and the frequency of contact with smokers at work. Small differences were observed in the responses between the two groups. Specifically, never smokers gave higher risk score to snus and nicotine compared to smokers, although both groups gave a fairly high risk score. Similar perceptions about the contribution of nicotine to smoking-related disease, the dependence potential of nicotine replacement therapies and electronic cigarettes and the origin of nicotine in replacement therapies and electronic cigarettes were observed in both groups. More never smokers compared to smokers supported the ban of nicotine use in electronic cigarettes, while more smokers supported the ban of flavors.

### 3.3. Comparison Between Physicians and Nurses

The results of the comparison between physicians and nurses are presented in Table 3. Physicians were older and had more working time than nurses. More nurses were current smokers compared to physicians. Physicians reported a better level of information about smoking. Both groups, however, reported mostly very or fairly high level of knowledge about smoking.

Significant differences were observed between the two groups in their responses. Physicians gave higher risk scores to conventional cigarettes and lower to nicotine replacement therapies and oral smoking cessation medications compared to nurses. They also gave a lower risk score to nicotine and a higher score to inhaled smoke. Nicotine was ranked fourth by physicians in terms of the risk among all components of smoking listed, while nurses ranked nicotine third, with a slightly higher risk score compared to inhaled smoke. More nurses considered extremely or very important the contribution of nicotine to the development of smoking-related diseases compared with physicians. More physicians than nurses considered that nicotine replacement therapies were associated with lower health risk compared to smoking. Both groups overestimated the smoking cessation rates of nicotine replacement therapies and thought that nicotine in these products is synthetically produced rather than extracted from the tobacco plant. A greater proportion of physicians compared to nurses would recommend the long-term use of nicotine replacement therapies, although the rates were very low in both groups. About one out of three participants in both groups had recommended electronic cigarette use to smokers in the past; however, more nurses compared to physicians would recommend electronic cigarettes as smoking substitutes for smokers who are not willing to use other smoking cessation aids. More nurses than physicians believed erroneously that electronic cigarettes contain chemical additives approved for inhalation and that established quality certificates exist. More physicians than nurses suggested bans on electronic cigarettes use in public places as well as bans in advertising. Finally, more physicians than nurses said they knew the EU legislation on electronic cigarettes, although the rate of knowledge in both groups was extremely low.

### 3.4. Responses by Cardiologists and Respiratory Physicians

The responses of cardiologists and respiratory physicians compared to all other healthcare professionals are presented in Table 4. Participants of the former subgroup were older and had more working time. They also had more frequent contact with smokers in their working environment and reported better level of information about smoking, while fewer were current smokers.

Significant differences were observed between the two groups in their responses. Cardiologists and respiratory physicians gave higher risk scores to conventional cigarettes and to electronic cigarettes, and marginally lower risk score (*p* = 0.049) to oral smoking cessation medications compared to all other healthcare professionals. They also gave a slightly higher risk score to inhaled smoke. Nicotine was ranked fourth among all smoking components listed by both groups. More than half of the participants of both groups considered the contribution of nicotine to smoking-related disease in general and to atherosclerosis as extremely or very important. The vast majority in both groups considered that nicotine replacement therapies were associated with lower health risk compared to smoking. Additionally, both groups overestimated the smoking cessation rates of nicotine replacement therapies and thought that nicotine in these products is synthetically produced rather than extracted from the tobacco plant. Fewer cardiologists and respiratory physicians compared to all other participants had recommended electronic cigarette use to smokers in the past and a lower proportion would recommend electronic cigarettes as smoking substitutes for smokers who are unable or unwilling to quit smoking with approved smoking cessation aids. A higher proportion of cardiologists and respiratory physicians compared to all other participants considered that electronic cigarettes were not effective in substituting smoking and proposed a ban on sales to youngsters, on use in public places and on advertising. More physicians than nurses suggested bans on electronic cigarettes use in public places as well as bans in advertising. Finally, more cardiologists and respiratory physicians said they knew the EU legislation on electronic cigarettes, although the rate of knowledge in both groups was very low.

### 3.5. Knowledge Score

To assess the knowledge about nicotine, nicotine replacement therapies and electronic cigarettes, a ”knowledge score“ was calculated for each participant. The correct answers to specific questions were scored with 1 point, while no point was given or deducted for wrong answers. Questions 12–20, 22–24, 27, 28 and 30 were used to generate the knowledge score (see Supplementary materials).

The total knowledge score for all participants was 7.7 ± 2.4 points out of a maximum of 16 points. Males had higher knowledge score than women (8.3 ± 2.3 *vs.* 7.2 ± 2.3, *p* < 0.001). Furthermore, physicians had higher knowledge score than nurses (8.1 ± 2.4 *vs.* 6.8 ± 2.0 respectively, *p* < 0.001). Significant differences were found among participants depending on the profession and their specialty (one-way ANOVA *p* < 0.001). General practitioners (9.1 ± 1.9), respiratory physicians (8.6 ± 2.4) and pediatricians (8.5 ± 2.5) had higher knowledge scores, while nurses had the lowest score. No association was found between the knowledge score and the frequency of contact with smokers at work or the self-perceived level of knowledge about smoking. Also, there were no differences between smokers (current and former) and never smokers. A statistically significant but weak correlation between the knowledge score and working time was found (Pearson's r = 0.153, *p* = 0.013). In multivariate analysis (linear regression) high knowledge score was associated with male gender (β = 0.755, 95% CI = 0.153–6.041, *p* = 0.014) and general practitioners (β = 1.780, 95% CI = 0.533–3.008, *p* = 0.004), pediatricians (β = 1.415, 95% CI = 0.231–2.599, *p* = 0.019) and respiratory physicians (β = 1.379, 95% CI = 0.437–2.321, *p* = 0.004).

## 4. Discussion

This study evaluated the knowledge and perceptions of healthcare professionals about nicotine, nicotine replacement therapies and electronic cigarettes. Participants had very frequent contact with smokers at work and felt they had high level of knowledge about smoking. However, an overestimation of the risks of nicotine was observed, with a significant proportion of participants considering that it has an extremely or very important contribution to the development of smoking-related diseases, including lung cancer and cancer in other organs, despite the fact that nicotine is not classified as a carcinogen. Additionally, a substantial proportion considered nicotine replacement therapies equally addictive to smoking. The negative perception about nicotine was evident from the fact that the majority would not recommend the long-term use of nicotine replacement therapies to smokers although guidelines from health organizations note that the risk from long-term use of nicotine is minimal and incomparable to continuous smoking [45,46,47]. The knowledge about electronic cigarettes was rather poor, with many thinking that they involve combustion and that their operating temperature is higher compared to the combustion temperature of a tobacco cigarette. Few participants were aware of the pending EU legislation on electronic cigarettes. A significant proportion suggested that appropriate regulation should include banning nicotine and flavors in electronic cigarettes, although studies have shown that they contribute significantly to the smoking cessation effort [49,50,51,52]. Many proposed that electronic cigarettes should be licensed as medications, despite the fact that they are not used as a therapeutic product by consumers [53]. Finally, more than 40% would not recommend electronic cigarettes to smokers unable or unwilling to quit smoking by using approved medications, despite the fact that recently the American Heart Association suggested that the effort to quit smoking with the use of electronic cigarettes should be supported in this population of smokers [54].

It was noteworthy that more than half of participants reported being current or former smokers. Smoking among healthcare professionals has been a long-standing issue. In fact, one of the first epidemiological studies highlighting the correlation between smoking and cancer was conducted in British physicians [55]. In past decades, there were even cigarette advertisements from physicians [56]. In the U.S., 40% of physicians smoked in 1959, with the rate falling to 21% in the 70s and 18% in the 80s [57,58,59]. A meta-analysis of 81 studies examined the prevalence of smoking among healthcare professionals of various countries [60]. The lowest smoking rates were observed in the U.S., UK and Australia while Greece had the highest rate with a smoking prevalence of 49%. Recently, a study of Greek nurses found that 32% of the participants were current and 14% were former smokers [61], while another study found high prevalence of smoking and lack of formal training on cessation counseling among healthcare professions students [62]. Herein, the smoking prevalence was similar to the above-mentioned studies. This is an important problem since healthcare professionals are role models for their patients and society, and the fact that they smoke compromises their potential to persuade their smoking patients to quit.

The differences in responses between smokers (current and former) and non-smokers were examined, assuming that personal experience with the smoking habit would differentiate their perceptions about nicotine and nicotine-containing products. Indeed we found that smokers gave a lower risk score to snus, while fewer supported the ban on the use of nicotine in electronic cigarettes compared with non-smokers. However, both groups overestimated the risk of nicotine and its contribution to smoking-related disease. Additionally, the two groups showed a similar deficit in their knowledge about electronic cigarettes.

Large differences in the responses between physicians and nurses were observed. The nurses felt less informed about smoking. As a result, they considered nicotine replacement therapies and oral medications more hazardous and the contribution of nicotine to smoking-related disease more important compared to physicians. Nurses had a lower knowledge score compared to physicians. Although some physician specialties (general practitioners, pediatricians and lung physicians) were associated with higher knowledge score than nurses, the score of knowledge was relatively low in the whole sample. Given that smoking prevalence is extremely high in Greece and counseling by nurses has been shown to increase the chance of quitting smoking [36], the present study clearly demonstrates the need for the more thorough education of nurses, but also of physicians, about smoking in order to counsel smokers more effectively.

Significant knowledge gaps were observed in the study sample of cardiologists and respiratory physicians. These physicians treat the most prevalent smoking-related disease conditions and are also responsible for the smoking cessation clinics in Greece. Thus, they are probably considered by smokers as the most reliable source of information about smoking and smoking cessation. There is no doubt that it is preferable for smokers to quit without the use of any aid or with approved smoking cessation medications. However, the survey questions assessed the approach to a specific subpopulation of smokers: those who are unable or unwilling to quit with currently approved methods. These smokers will most likely maintain their smoking habit for a long time; thus, it is important that they receive reliable and unbiased information not only about electronic cigarettes but also about the long term use of nicotine replacement therapies, because these products could have an important role in significantly reducing their health risk.

Misconceptions about the relative risk of alternative nicotine-containing products compared to smoking have also been observed in the general public. Kiviniemi and Kozlowski recently reported that only 9% of a nationally representative sample of U.S. adults perceived smokeless tobacco products to be less harmful than smoking [63]. One of the reasons for such perceptions is related to the “not safe” (in absolute terms) argument, which has been one of the central themes of tobacco control efforts and of various scientific organizations but has been criticized as poor-quality health information [64]. Herein a significant proportion considered electronic cigarettes equally or even more harmful than smoking. This is expected, since there are scientific societies which have proposed to ban alternative to smoking products such as electronic cigarettes [65], despite emerging evidence showing clinical benefit for smokers who switch to such products [66,67]. The basis for such proposals has been the precautionary principle (based on the lack of long-term studies) which, however, seems to be applied erroneously [68]. Additionally, there is a strong ideological opposition against the use of products containing nicotine [69]. Thus, there are confusing reports about the benefits and risks of products such as electronic cigarettes [68,70], which are likely influencing the perceptions of healthcare professionals. Moreover, participants were not aware of the pending EU legislation which, once implemented in May 2016, will create some quality standards through a proposed testing regime, will define limits on nicotine content and require consistent nicotine delivery, and will enforce the registration of all products available in the market. Thus, many healthcare professionals may think that electronic cigarettes will remain completely unregulated, creating uncertainty about their safety, quality and efficacy. Finally, there is a well-established lack of education especially at the undergraduate level in medical and nursing schools about smoking and smoking cessation [71,72]. Potential improvements in healthcare professionals’ understanding about, and approach to, smoking alternatives are expected through better personal education (undergraduate and postgraduate) and through a reconsideration of the scientific community positioning towards tobacco harm reduction.

One of the limitations of the study is the applicability of the findings to all healthcare professionals in Greece and abroad. Obviously, the study recruited a convenient sample of healthcare professionals, so the findings should be interpreted with caution. However, the observations are not very different from similar surveys (mainly on physicians) evaluating perceptions for electronic cigarettes or other tobacco harm reduction products. Evidence from a study in Norway showed that healthcare professionals overestimate the adverse effects of another tobacco harm reduction product, snus [42]. Another study among Swedish and British general practitioners found that participants had limited knowledge about nicotine in tobacco products and pharmaceutical nicotine [37]. Finally, two recent studies found that only 30%–35% of surveyed physicians from the United States have recommended electronic cigarettes to smokers [73,74]. Our findings are consistent with all these studies but present the views of a more generalized sample of healthcare professionals, including nurses and several physician specialties. However, future research recruiting a random sample of healthcare professionals would be valuable to further explore this issue. Another limitation of the study is the 30.3% response rate, which appears to be low. However, this is considered acceptable [75,76] and comparable to other physician email surveys. Dykema *et al.* had a response rate of 7.4% despite using a financial incentive for participation [77]. Pepper *et al.* evaluated the perceptions of physicians who treat adolescents about electronic cigarettes and had a response rate of 28% [78], while Kandra *et al.* performed a similar study among North Carolina physicians and had a response rate of 31% [74]. General practitioners represented only 6.5% of the study sample. Although for most countries smokers are more likely to visit general practitioners for health-related issues, including smoking, the situation in Greece is different. Patients can visit specialist physicians in primary care on their own initiative (without the need to be referred by a general practitioner), and all medical specialties included in this study are available in primary care centers in Greece. Additionally, it is very common for specialists to have private offices and work as primary healthcare providers. Thus, the low proportion of general practitioners is not surprising. Finally, while a knowledge score was calculated, it does not cover all aspects of scientific information about nicotine and electronic cigarettes, it was a secondary objective of the study and was calculated to provide a rough generalized estimate of knowledge based on the specific questions of the survey. It is not proposed as a validated score to determine such knowledge in future studies.

## 5. Conclusions

Greek healthcare professionals have a significant knowledge deficit about nicotine, nicotine replacement therapies and electronic cigarettes. This is expected to have negative implications in providing appropriate and reliable counseling to smokers. A substantial proportion of the participants were reluctant to recommend nicotine-containing pharmaceutical products or electronic cigarettes as long-term smoking substitutes to smokers unwilling or unable to quit with currently approved methods. The continuous elevation in awareness and use of electronic cigarettes by smokers is expected to increase their demand for appropriate information and advice about these products. To fulfill their role as reliable providers of factual information and advice to smokers, healthcare professionals need to be properly educated and to closely follow the scientific evidence on nicotine and electronic cigarettes, which is expanding at a very rapid rate in recent years.

## Figures and Tables

**Table 1:** Characteristics and responses of all survey participants.

Participant Characteristics and Responses (*n* = 262)	Mean (SD) or *n* (%)
**Gender**	
Males	138 (52.7%)
Females	124 (47.3%)
**Age (Years)**	39 (9)
**Profession**	
Nurse	77 (29.4%)
Physician	185 (70.6%)
General practitioner	17 (6.5%)
Cardiologist	53 (20.2%)
Dentist	32 (12.2%)
Internist	32 (12.2%)
Pediatrician	17 (6.5%)
Respiratory physician	34 (13.0%)
**Working Sector**	
Public sector	162 (61.8%)
Private sector	100 (38.2%)
**Working time**	12 (8)
**Contact with smokers in the working environment**	
Daily	222 (84.7%)
At least 3 days per week	15 (5.7%)
1–2 days per weekly	6 (2.3%)
<1 day per week	18 (6.9%)
**Smokers**	80 (30.5%)
Consider quitting *	48 (60.0%)
**Former smokers**	59 (22.5%)
**Smoking cessation method for former smokers**	
No aid	47 (17.9%)
Nicotine replacement therapy	1 (0.4%)
Oral medications	3 (1.1%)
Electronic cigarette	7 (2.7%)
Other	1 (0.4%)
**Self-perceived level of knowledge about smoking**	
Very high	99 (37.8%)
Fairly high	116 (44.3%)
Moderate	43 (16.4%)
Low	4 (1.5%)
**Risk score for products**	
Tobacco cigarettes	9.3 (1.1)
Snus	8.3 (1.7)
Electronic cigarettes	5.9 (2.4)
Nicotine replacement therapy	4.0 (2.3)
Oral medications	4.2 (2.2)
**Risk score for smoking components**	
Nicotine	8.0 (2.1)
Inhaled smoke	8.8 (1.4)
Carbon monoxide	9.1 (1.4)
Tar	9.5 (0.9)
Tobacco	6.7 (2.4)
**Contribution of nicotine to smoking-related disease**	
Extremely important	63 (24.0%)
Very important	106 (40.5%)
Important	55 (21.0%)
Less important	26 (9.9%)
Minimal	12 (4.6%)
**Contribution of nicotine to lung cancer**	
Extremely important	63 (24.8%)
Very important	86 (32.8%)
Important	49 (18.7%)
Less important	37 (14.1%)
Minimal	25 (9.5%)
**Contribution of nicotine to cancer in other organs**	
Extremely important	28 (10.7%)
Very important	88 (33.6%)
Important	71 (27.1%)
Less important	53 (20.2%)
Minimal	22 (8.4%)
**Contribution of nicotine to atherosclerosis**	
Extremely important	68 (26.0%)
Very important	105 (40.1%)
Important	50 (19.1%)
Less important	28 (10.7%)
Minimal	11 (4.2%)
**Risk of nicotine replacement therapies compared to smoking**	
Higher	3 (1.1%)
Equal	42 (16.0%)
Lower	200 (76.3%)
Do not know	17 (6.5%)
**Dependence potential of nicotine replacement therapies compared to smoking**	
Higher	3 (1.1%)
Equal	84 (32.1%)
Lower	146 (55.7%)
Do not know	29 (11.1%)
**Success rate of nicotine replacement therapies in smoking cessation at 1 year**	
>50%	12 (4.6%)
30%–50%	63 (24.0%)
10%–30%	126 (48.1%)
<10%	61 (23.3%)
**Origin of nicotine in nicotine replacement therapies**	
Tobacco-extracted	42 (16.0%)
Synthetically-produced	220 (84.0%)
**Safe to use nicotine replacement therapies for >6 months as substitutes to smoking?**	
Yes	65 (24.8%)
No	197 (75.2%)
**Recommend the long-term (>6 months) use of nicotine replacement therapies for those who cannot reduce or quit smoking with short-term use?**	
Yes	55 (21.0%)
No	207 (79.0%)
**Have you ever recommended e-cigarettes to smokers?**	
Yes	87 (33.2%)
No	175 (66.8%)
**Risk of e-cigarettes compared to smoking**	
Higher	4 (1.5%)
Equal	60 (22.9%)
Lower	174 (66.4%)
Do not know	24 (9.2%)
**Dependence potential of e-cigarettes compared to smoking**	
Higher	6 (2.3%)
Equal	112 (42.7%)
Lower	120 (45.8%)
Do not know	24 (9.2%)
**Origin of nicotine in e-cigarettes**	
Tobacco-extracted	34 (13.0%)
Synthetically-produced	228 (87.0%)
**Would you recommend e-cigarettes to smokers who refuse to take medications to quit?**	
Yes	147 (56.1%)
No	115 (43.9%)
**Would you recommend e-cigarettes to smokers who failed to quit with other methods?**	
Yes	156 (59.5%)
No	106 (40.5%)
**Which of the following are correct concerning e-cigarettes?**	
They contain tobacco	19 (7.3%)
There is combustion	93 (35.5%)
E-liquid ingredients are approved for inhalation	172 (65.6%)
Working temperature is lower than in tobacco cigarettes	97 (37.0%)
They have official quality certificates	66 (25.2%)
There are e-cigarettes without nicotine	152 (58.0%)
**Do you consider e-cigarettes effective in substituting smoking?**	
Yes	134 (51.1%)
No	128 (48.9%)
**What do you think regulation on e-cigarettes should include?**	
Available only through prescription	114 (43.5%)
Ban on nicotine	115 (43.9%)
No flavors	52 (19.8%)
Ban on the sales to youngsters	193 (73.7%)
Ban on the use in public places	74 (28.2%)
They should be licensed as medications	113 (43.1%)
They should be sold only in pharmacies	100 (38.2%)
Product variability should be reduced	64 (24.4%)
Advertising should be banned	104 (39.7%)
There should be a warning that they are equally harmful to Smoking	110 (42.0%)
**Do you know the European Union regulatory framework on e-cigarettes?**	
Yes	38 (14.5%)
No	223 (85.1%)

* Percentage represents proportion of smokers.

**Table 2:** Comparison of survey responses between smokers (current and former) and never-smokers.

Participant Characteristics and Responses (*n* = 262)	Smokers (Current and Former)	Never Smokers	*p*
	139 (53.1%)	123 (46.9%)	
**Gender**			
Males	69 (49.6%)	55 (44.7%)	0.426
Females	70 (50.4%)	68 (55.3%)	
**Age (Years)**	39 (9.0%)	39 (8.0%)	0.462
**Profession**			
Nurse	46 (33.1%)	31 (25.2%)	0.162
Physician	93 (66.9%)	92 (74.8%)	
General practitioner	10 (7.2%)	7 (5.7%)	
Cardiologist	24 (17.3%)	29 (23.6%)	
Dentist	18 (12.9%)	14 (11.4%)	
Internist	14 (10.1%)	18 (14.6%)	
Pediatrician	9 (6.5%)	8 (6.5%)	
Respiratory physician	18 (12.9%)	16 (13.0%)	
**Working sector**			
Public sector	88 (63.3%)	74 (60.2%)	
Private sector	51 (36.7%)	49 (39.8%)	
**Working time**	13 (8)	12 (8)	0.710
**Contact with smokers in the working environment**			
Daily	117 (84.2%)	105 (86.1%)	0.919
At least 3 days per week	8 (5.8%)	7 (5.7%)	
1–2 days per weekly	3 (2.2%)	3 (2.5%)	
<1 day per week	11 (7.9%)	7 (5.7%)	
**Smoking cessation method for former smokers**			
No aid	47 (17.9%)		
Nicotine replacement therapy	1 (0.4%)		
Oral medications	3 (1.1%)		
Electronic cigarette	7 (2.7%)		
Other	1 (0.4%)		
**Self-perceived level of knowledge about smoking**			
Very high	57 (41.0%)	42 (34.1%)	0.080
Fairly high	63 (45.3%)	53 (43.1%)	
Moderate	19 (13.7%)	21 (19.5%)	
Low	0 (0.0%)	4 (3.3%)	
**Risk score for products**			
Tobacco cigarettes	9.3 (1.2)	9.4 (1.0)	0.252
Snus	7.8 (1.9)	8.9 (1.2)	<0.001
Electronic cigarettes	5.9 (2.4)	5.9 (2.5)	0.923
Nicotine replacement therapy	4.3 (2.2)	4.5 (2.3)	0.529
Oral medications	4.3 (2.3)	4 (2.1)	0.201
**Risk score for smoking components**			
Nicotine	7.7 (2.4)	8.3 (1.8)	0.012
Inhaled smoke	8.8 (1.4)	8.9 (1.4)	0.577
Carbon monoxide	9.0 (1.5)	9.2 (1.2)	0.101
Tar	9.6 (0.8)	9.5 (1.0)	0.386
Tobacco	6.7 (2.5)	6.7 (2.4)	0.773
**Contribution of nicotine to smoking-related disease**			
Extremely important	29 (20.9%)	34 (27.6%)	0.679
Very important	57 (41.0%)	49 (39.8%)	
Important	31 (22.3%)	24 (19.5%)	
Less important	16 (11.5%)	10 (8.1%)	
Minimal	6 (4.3%)	6 (4.9%)	
**Contribution of nicotine to lung cancer**			
Extremely important	32 (23.0%)	33 (26.8%)	0.894
Very important	48 (34.5%)	38 (30.9%)	
Important	26 (18.7%)	23 (18.7%)	
Less important	21 (15.1%)	16 (13.0%)	
Minimal	12 (8.6%)	13 (10.6%)	
**Contribution of nicotine to cancer in other organs**			
Extremely important	15 (10.8%)	13 (10.6%)	0.880
Very important	46 (33.1%)	42 (34.1%)	
Important	37 (26.6%)	34 (27.6%)	
Less important	31 (22.3%)	22 (17.9%)	
Minimal	10 (7.2%)	12 (9.8%)	
**Contribution of nicotine to atherosclerosis**			
Extremely important	39 (28.1%)	29 (23.6%)	0.299
Very important	60 (43.2%)	45 (36.6%)	
Important	20 (14.4%)	30 (24.4%)	
Less important	15 (10.8%)	13 (10.6%)	
Minimal	5 (3.6%)	6 (4.9%)	
**Risk of nicotine replacement therapies compared to smoking**			
Higher	1 (0.7%)	2 (1.6%)	0.302
Equal	27 (19.4%)	15 (12.2%)	
Lower	104 (74.8%)	96 (78.0%)	
Do not know	7 (5.0%)	10 (8.1%)	
**Dependence potential of nicotine replacement therapies compared to smoking**			
Higher	1 (0.7%)	2 (1.6%)	0.702
Equal	48 (34.5%)	36 (29.3%)	
Lower	74 (53.2%)	72 (58.5%)	
Do not know			
**Success rate of nicotine replacement therapies in smoking cessation at 1 year**			
>50%	5 (3.6%)	7 (5.7%)	0.145
30%–50%	28 (20.1%)	35 (28.5%)	
10%–30%	67 (48.2%)	59 (48.0%)	
<10%	39 (28.1%)	22 (17.9%)	
**Origin of nicotine in nicotine replacement therapies**			
Tobacco-extracted	22 (15.8%)	20 (16.3%)	0.924
Synthetically-produced	117 (84.2%)	103 (83.7%)	
**Safe to use nicotine replacement therapies for >6 months as substitutes to smoking?**			
Yes	35 (25.2%)	30 (24.4%)	0.883
No	104 (74.8%)	93 (75.6%)	
**Recommend the long-term (>6 months) use of nicotine replacement therapies for those who cannot reduce or quit smoking with short-term use?**			
Yes	26 (18.7%)	29 (23.6%)	0.334
No	113 (81.3%)	94(76.4%)	
**Have you ever recommended e-cigarettes to smokers?**			
Yes	46 (33.1%)	41 (33.3%)	0.967
No	93 (66.9%)	82 (66.7%)	
**Risk of e-cigarettes compared to smoking**			
Higher	1 (0.7%)	3(1.1%)	0.451
Equal	36 (25.9%)	24 (19.5%)	
Lower	90 (64.7%)	84 (68.3%)	
Do not know	12 (8.6%)	12 (9.8%)	
**Dependence potential of e-cigarettes compared to smoking**			
Higher	4 (2.9%)	2 (1.6%)	0.649
Equal	63 (45.3%)	49 (39.8%)	
Lower	61 (43.9%)	59 (48.0%)	
Do not know	11 (7.9%)	13 (10.6%)	
**Origin of nicotine in e-cigarettes**			
Tobacco-extracted	20 (14.4%)	14 (11.4%)	0.470
Synthetically-produced	119 (85.6%)	109 (88.6%)	
**Would you recommend e-cigarettes to smokers who refuse to take medications to quit?**			
Yes	81 (58.3%)	66 (53.7%)	0.453
No	58 (41.7%)	57 (46.3%)	
**Would you recommend e-cigarettes to smokers who failed to quit with other methods?**			
Yes	82 (59.0%)	74 (60.2%)	0.847
No	57 (41.0%)	49 (39.8%)	
**Which of the following are correct concerning e-cigarettes?**			
They contain tobacco	11 (7.9%)	8 (6.5%)	0.661
There is combustion	51 (36.7%)	42 (34.1%)	0.668
E-liquid ingredients are approved for inhalation	92 (66.2%)	80 (65.0%)	0.845
Working temperature is lower than in tobacco cigarettes	49 (35.3%)	48 (39.0%)	0.528
They have official quality certificates	36 (25.9%)	30 (24.4%)	0.779
There are e-cigarettes without nicotine	86 (61.9%)	66 (53.7%)	0.179
**Do you consider e-cigarettes effective in substituting smoking?**			
Yes	69 (49.6%)	65 (52.8%)	0.604
No	70 (50.4%)	58 (47.2%)	
**What do you think regulation on e-cigarettes should include?**			
Available only through prescription	56 (40.3%)	61 (49.6%)	0.131
Ban on nicotine	48 (34.5%)	67 (54.5%)	0.001
No flavors	35 (25.2%)	17 (13.8%)	0.021
Ban on the sales to youngsters	104 (74.8%)	89 (72.4%)	0.652
Ban on the use in public places	33 (23.7%)	41 (33.3%)	0.085
They should be licensed as medications	56 (40.3%)	57 (46.3%)	0.323
They should be sold only in pharmacies	46 (33.1%)	54 (43.9%)	0.072
Product variability should be reduced	36 (25.9%)	28 (22.8%)	0.556
Advertising should be banned	53 (38.1%)	51 (41.5%)	0.582
There should be a warning that they are equally harmful to smoking	62 (44.6%)	48 (39.0%)	0.361
**Do you know the European Union regulatory framework on e-cigarettes?**			
Yes	17 (12.2%)	21 (12.2%)	0.255
No	122 (87.8%)	101 (82.8%)	

**Table 3:** Comparison of survey responses between physicians and nurses.

Participant Characteristics and Responses (*n* = 262)	Physicians	Nurses	*p*
	185 (70.6%)	77 (29.4%)	
**Gender**			
Males	109 (58.9%)	15 (19.5%)	<0.001
Females	76 (41.1%)	62 (81.5%)	
**Age**	41 (8)	33 (6)	<0.001
**Working sector**			
Public sector	98 (53.0%)	64 (83.1%)	<0.001
Private sector	87 (47.0%)	13 (16.9%)	
**Working time**	14 (9)	9 (5)	<0.001
**Contact with smokers in the working environment**			
Daily	159 (85.9%)	63 (82.9%)	0.676
At least 3 days per week	11 (5.9%)	4 (5.3%)	
1–2 days per weekly	3 (1.6%)	3 (3.9%)	
<1 day per week	12 (6.5%)	6 (7.9%)	
**Smokers**	44 (23.8%)	36 (46.8%)	<0.001
Consider quitting *	25 (56.8%)	23 (63.9%)	0.521
**Former smokers**	49 (34.8%)	10 (24.4%)	0.212
**Smoking cessation method for former smokers**			
No aid	39 (79.6%)	8 (80.0%)	0.792
Nicotine replacement therapy	1 (2.0%)	0 (0.0%)	
Oral medications	3 (6.1%)	0 (0.0%)	
Electronic cigarette	5 (10.2%)	2 (20.0%)	
Other	1 (2.0%)	0 (0.0%)	
**Self-perceived level of knowledge about smoking**			
Very high	80 (43.2%)	19 (24.7%)	0.006
Fairly high	79 (42.7%)	37 (48.1%)	
Moderate	25 (13.5%)	18 (23.4%)	
Low	1 (0.5%)	3 (3.9%)	
**Risk score for products**			
Tobacco cigarettes	9.5 (0.9)	9.0 (1.7)	0.008
Snus	8.3 (1.7)	8.3 (1.6)	0.952
Electronic cigarettes	6.0 (2.4)	5.7 (2.5)	0.240
Nicotine replacement therapy	4.2 (2.2)	5.0 (2.2)	0.005
Oral medications	3.8 (2.1)	5.1 (2.3)	<0.001
**Risk score for smoking components**			
Nicotine	7.8 (2.3)	8.5 (1.6)	0.005
Inhaled smoke	9.0 (1.3)	8.4 (1.5)	0.007
Carbon monoxide	9.1 (1.4)	9.1 (1.3)	0.794
Tar	9.6 (0.9)	9.5 (0.9)	0.496
Tobacco	6.6 (2.4)	6.9 (2.4)	0.401
**Contribution of nicotine to smoking-related disease**			
Extremely important	42 (22.7%)	21 (27.3%)	0.011
Very important	65 (35.1%)	41 (53.2%)	
Important	45 (24.3%)	10 (13.0%)	
Less important	23 (12.4%)	3 (3.9%)	
Minimal	10 (5.4%)	2 (2.6%)	
**Contribution of nicotine to lung cancer**			
Extremely important	43 (23.2%)	22 (28.6%)	<0.001
Very important	47 (25.4%)	39 (50.6%)	
Important	38 (20.5%)	11 (14.3%)	
Less important	34 (18.4%)	3 (3.9%)	
Minimal	23 (12.4%)	2 (2.6%)	
**Contribution of nicotine to cancer in other organs**			
Extremely important	21 (11.4%)	7 (9.1%)	0.007
Very important	51 (27.6%)	37 (48.1%)	
Important	50 (27.0%)	21 (27.3%)	
Less important	43 (23.2%)	10 (13.0%)	
Minimal	20 (10.8%)	2 (2.6%)	
**Contribution of nicotine to atherosclerosis**			
Extremely important	45 (24.3%)	23 (29.9%)	<0.001
Very important	62 (33.5%)	43 (55.8%)	
Important	42 (22.7%)	8 (10.4%)	
Less important	26 (14.1%)	2 (2.6%)	
Minimal	10 (5.4%)	1 (1.3%)	
**Risk of nicotine replacement therapies compared to smoking**			
Higher	2 (1.1%)	1 (1.3%)	0.044
Equal	23 (12.4%)	19 (24.7%)	
Lower	150 (81.1%)	50 (64.9%)	
Do not know	10 (5.4%)	7 (9.1%)	
**Dependence potential of nicotine replacement therapies compared to smoking**			
Higher	3 (1.6%)	0 (0.0%)	0.006
Equal	53 (28.6%)	31 (40.3%)	
Lower	114 (61.6%)	32 (41.6%)	
Do not know	15 (8.1%)	14 (18.2%)	
**Success rate of nicotine replacement therapies in smoking cessation at 1 year**			
>50%	8 (4.3%)	4 (5.2%)	0.991
30%–50%	45 (24.3%)	18 (23.4%)	
10%–30%	89 (48.1%)	37 (48.1%)	
<10%	43 (23.2%)	18 (23.4%)	
**Origin of nicotine in nicotine replacement therapies**			
Tobacco-extracted	32 (17.3%)	10 (13.0%)	0.386
Synthetically-produced	153 (82.7%)	67 (87.0%)	
**Safe to use nicotine replacement therapies for >6 months as substitutes to smoking?**			
Yes	53 (28.6%)	12 (15.6%)	0.026
No	132 (71.4%)	65 (84.4%)	
**Recommend the long-term (>6 months) use of nicotine replacement therapies for those who cannot reduce or quit smoking with short-term use?**			
Yes	45 (24.3%)	10 (13.0%)	0.040
No	140 (75.7%)	67 (87.0%)	
**Have you ever recommended e-cigarettes to smokers?**			
Yes	58 (31.4%)	29 (37.7%)	0.323
No	127 (68.6%)	48 (62.3%)	
**Risk of e-cigarettes compared to smoking**			
Higher	4 (2.2%)	0 (0.0%)	0.542
Equal	43 (23.2%)	17 (22.1%)	
Lower	120 (64.9%)	54 (70.1%)	
Do not know	18 (9.7%)	6 (7.8%)	
**Dependence potential of e-cigarettes compared to smoking**			
Higher	5 (2.7%)	1 (1.3%)	0.212
Equal	75 (40.5%)	37 (48.1%)	
Lower	91 (49.2%)	29 (37.7%)	
Do not know	14 (7.6%)	10 (13.0%)	
**Origin of nicotine in e-cigarettes**			
Tobacco-extracted	25 (13.5%)	9 (11.7%)	0.689
Synthetically-produced	160 (86.5%)	68 (88.3%)	
**Would you recommend e-cigarettes to smokers who refuse to take medications to quit?**			
Yes	95 (51.4%)	52 (67.5%)	0.016
No	90 (48.6%)	25 (32.5%)	
**Would you recommend e-cigarettes to smokers who failed to quit with other methods?**			
Yes	104 (56.2%)	52 (67.5%)	0.089
No	81 (43.8%)	25 (32.5%)	
**Which of the following are correct concerning e-cigarettes?**			
They contain tobacco	11 (5.9%)	8 (10.4%)	0.206
There is combustion	68 (36.8%)	25 (32.5%)	0.509
E-liquid ingredients are approved for inhalation	114 (61.6%)	58 (75.3%)	0.033
Working temperature is lower than in tobacco cigarettes	72 (38.9%)	25 (32.5%)	0.325
They have official quality certificates	40 (21.6%)	26 (39.4%)	0.039
There are e-cigarettes without nicotine	109 (58.9%)	43 (55.8%)	0.646
**Do you consider e-cigarettes effective in substituting smoking?**			
Yes	91 (49.2%)	43 (55.8%)	0.326
No	94 (50.8%)	34 (44.2%)	
**What do you think regulation on e-cigarettes should include?**			
Available only through prescription	81 (43.8%)	36 (46.8%)	0.660
Ban on nicotine	76 (41.1%)	39 (50.6%)	0.155
No flavors	38 (20.5%)	14 (18.2%)	0.663
Ban on the sales to youngsters	141 (76.2%)	52 (67.5%)	0.146
Ban on the use in public places	61 (33.0%)	13 (16.9%)	0.008
They should be licensed as medications	84 (45.4%)	29 (37.7%)	0.249
They should be sold only in pharmacies	75 (40.5%)	25 (32.5%)	0.220
Product variability should be reduced	46 (24.9%)	18 (23.4%)	0.798
Advertising should be banned	81 (43.8%)	23 (29.9%)	0.036
There should be a warning that they are equally harmful to smoking	73 (39.5%)	37 (48.1%)	0.199
**Do you know the European Union regulatory framework on e-cigarettes?**			
Yes	34 (18.5%)	4 (5.2%)	0.006
No	150 (81.5%)	73 (94.8%)	

* Percentages represent proportion of smokers.

**Table 4:** Survey responses of cardiologists and respiratory physicians compared to all other participants.

Participant Characteristics and Responses (*n* = 262)	Cardiologists and Respiratory Physicians	All Others	*p*
	87 (33.2%)	175 (66.8%)	
**Gender**			
Males	58 (66.7%)	66 (37.7%)	<0.001
Females	29 (33.3%)	109 (62.3%)	
**Age**	43 (9)	37 (8)	<0.001
**Working sector**			
Public sector	49 (56.3%)	113 (64.6%)	0.195
Private sector	38 (43.7%)	62 (35.4%)	
**Working time**	15 (9)	11 (7)	0.001
**Contact with smokers in the working environment**			
Daily	82 (94.3%)	140 (80.5%)	0.013
At least 3 days per week	4 (4.6%)	11 (6.3%)	
1–2 days per weekly	0 (0.0%)	6 (3.4%)	
<1 day per week	1 (1.1%)	17 (9.8%)	
**Smokers**	18 (20.7%)	62 (35.4%)	0.015
Consider quitting *	12 (66.7%)	36 (58.1%)	0.512
**Former smokers**	24 (34.8%)	35 (31.0%)	0.594
**Smoking cessation method for former smokers**			
No aid	19 (79.2%)	28 (80.0%)	0.017
Nicotine replacement therapy	1 (4.2%)	0 (0.0%)	
Oral medications	3 (12.5%)	0 (0.0%)	
Electronic cigarette	0 (0.0%)	7 (20.0%)	
Other	1 (4.2%)	0 (0.0%)	
**Self-perceived level of knowledge about smoking**			
Very high	46 (52.9%)	53 (30.3%)	0.003
Fairly high	30 (34.5%)	86 (49.1%)	
Moderate	11 (12.6%)	32 (18.3%)	
Low	0 (0.0%)	4 (2.3%)	
**Risk score for products**			
Tobacco cigarettes	9.6 (0.7)	9.2 (1.2)	<0.001
Snus	8.3 (1.6)	8.3 (1.7)	0.921
Electronic cigarettes	6.7 (2.4)	5.6 (2.4)	<0.001
Nicotine replacement therapy	4.1 (2.5)	4.5 (2.1)	0.292
Oral medications	3.8 (2.4)	4.3 (2.1)	0.049
**Risk score for smoking components**			
Nicotine	7.8 (2.3)	8.1 (2.1)	0.327
Inhaled smoke	9.2 (1.2)	8.6 (1.5)	0.002
Carbon monoxide	9.1 (1.4)	9.1 (1.3)	0.725
Tar	9.6 (0.9)	9.5 (0.9)	0.225
Tobacco	6.5 (2.5)	6.8 (2.4)	0.292
**Contribution of nicotine to smoking-related disease**			
Extremely important	18 (20.7%)	45 (25.7%)	0.305
Very important	32 (36.8%)	74 (42.3%)	
Important	25 (28.7%)	30 (17.1%)	
Less important	8 (9.2%)	18 (10.3%)	
Minimal	4 (4.6%)	8 (4.6%)	
**Contribution of nicotine to lung cancer**			
Extremely important	20 (23.0%)	45 (25.7%)	0.130
Very important	23 (26.4%)	63 (36.0%)	
Important	15 (17.2%)	34 (19.4%)	
Less important	17 (19.5%)	20 (11.4%)	
Minimal	12 (13.8%)	13 (7.4%)	
**Contribution of nicotine to cancer in other organs**			
Extremely important	11 (12.6%)	17 (9.7%)	0.186
Very important	29 (33.3%)	59 (33.7%)	
Important	17 (19.5%)	54 (30.9%)	
Less important	19 (21.8%)	34 (19.4%)	
Minimal	11 (6.3%)	11 (12.6%)	
**Contribution of nicotine to atherosclerosis**			
Extremely important	25 (28.7%)	43 (24.6%)	0.040
Very important	25 (28.7%)	80 (45.7%)	
Important	18 (20.7%)	32 (18.3%)	
Less important	15 (17.2%)	13 (7.4%)	
Minimal	4 (4.6%)	7 (4.0%)	
**Risk of nicotine replacement therapies compared to smoking**			
Higher	2 (2.3%)	1 (0.6%)	0.135
Equal	9 (10.3%)	33 (18.9%)	
Lower	72 (82.8%)	128 (73.1%)	
Do not know	4 (4.6%)	13 (7.4%)	
**Dependence potential of nicotine replacement therapies compared to smoking**			
Higher	2 (2.3%)	1 (0.6%)	0.137
Equal	27 (31.0%)	57 (32.6%)	
Lower	53 (60.9%)	93 (53.1%)	
Do not know	5 (5.7%)	24 (13.7%)	
**Success rate of nicotine replacement therapies in smoking cessation at 1 year**			
>50%	5 (5.7%)	7 (4.0%)	0.660
30%–50%	24 (27.6%)	39 (22.3%)	
10%–30%	38 (43.7%)	88 (50.3%)	
<10%	20 (23.0%)	41 (23.4%)	
**Origin of nicotine in nicotine replacement therapies**			
Tobacco-extracted	16 (18.4%)	26 (14.9%)	0.463
Synthetically-produced	71 (81.6%)	149 (85.1%)	
**Safe to use nicotine replacement therapies for >6 months as substitutes to smoking?**			
Yes	26 (29.9%)	39 (22.3%)	0.180
No	61 (70.1%)	136 (77.7%)	
**Recommend the long-term (>6 months) use of nicotine replacement therapies for those who cannot reduce or quit smoking with short-term use?**			
Yes	23 (26.4%)	32 (18.3%)	0.127
No	64 (73.6%)	143 (81.7%)	
**Have you ever recommended e-cigarettes to smokers?**			
Yes	21 (24.1%)	66 (37.7%)	0.028
No	66 (75.9%)	109 (62.3%)	
**Risk of e-cigarettes compared to smoking**			
Higher	4 (4.6%)	0 (0.0%)	0.006
Equal	26 (29.9%)	34 (19.4%)	
Lower	50 (57.5%)	124 (70.9%)	
Do not know	7 (8.0%)	17 (9.7%)	
**Dependence potential of e-cigarettes compared to smoking**			
Higher	3 (3.4%)	3 (1.7%)	0.160
Equal	44 (50.6%)	68 (38.9%)	
Lower	35 (40.2%)	85 (48.6%)	
Do not know	5 (5.7%)	19 (10.9%)	
**Origin of nicotine in e-cigarettes**			
Tobacco-extracted	12 (13.8%)	22 (12.6%)	0.782
Synthetically-produced	75 (86.2%)	153 (87.4%)	
**Would you recommend e-cigarettes to smokers who refuse to take medications to quit?**			
Yes	34 (39.1%)	113 (64.6%)	<0.001
No	53 (60.9%)	62 (35.4%)	
**Would you recommend e-cigarettes to smokers who failed to quit with other methods?**			
Yes	41 (47.1%)	115 (65.7%)	0.004
No	46 (52.9%)	60 (34.3%)	
**Which of the following are correct concerning e-cigarettes?**			
They contain tobacco	5 (5.7%)	14 (8.0%)	0.508
There is combustion	32 (36.8%)	61 (34.9%)	0.759
E-liquid ingredients are approved for inhalation	53 (60.9%)	119 (68.0%)	0.256
Working temperature is lower than in tobacco cigarettes	34 (39.1%)	63 (36.0%)	0.627
They have official quality certificates	16 (18.4%)	50 (28.6%)	0.074
There are e-cigarettes without nicotine	55 (63.2%)	97 (55.4%)	0.229
**Do you consider e-cigarettes effective in substituting smoking?**			
Yes	35 (40.2%)	99 (56.6%)	0.013
No	52 (59.8%)	76 (43.4%)	
**What do you think regulation on e-cigarettes should include?**			
Available only through prescription	35 (40.2%)	82 (46.9%)	0.310
Ban on nicotine	30 (34.5%)	85 (48.6%)	0.030
No flavors	20 (23.0%)	32 (18.3%)	0.369
Ban on the sales to youngsters	72 (82.8%)	121 (69.1%)	0.018
Ban on the use in public places	38 (43.7%)	36 (20.6%)	<0.001
They should be licensed as medications	43 (49.4%)	70 (40.0%)	0.147
They should be sold only in pharmacies	34 (39.1%)	66 (37.7%)	0.830
Product variability should be reduced	24 (27.6%)	40 (22.9%)	0.401
Advertising should be banned	45 (51.7%)	59 (33.7%)	0.005
There should be a warning that they are equally harmful to smoking	40 (46.0%)	70 (40.0%)	0.356
**Do you know the European Union regulatory framework on e-cigarettes?**			
Yes	23 (26.4%)	15 (8.6%)	<0.001
No	64 (73.6%)	159 (91.4%)	

* Percentages represent proportion of smokers.

## Supplementary Materials

The following are available online at www.mdpi.com/1660-4601/13/5/514/s1, Survey Questionnaire.
