# Supplementary material for: Knowledge and Perceptions about Nicotine, Nicotine Replacement Therapies and Electronic Cigarettes among Healthcare Professionals in Greece
Source: Int J Environ Res Public Health. 2016 May 20;13(5):514. doi: 10.3390/ijerph13050514 (PMC4881139; doi:10.3390/ijerph13050514)
Supplement: Supplementary file 1 [file ijerph-13-00514-s001.pdf]

Supplementary Materials: Knowledge and Perceptions about Nicotine, Nicotine Replacement Therapies and Electronic Cigarettes among Healthcare Professionals in Greece

Anastasia Moysidou, Konstantinos E. Farsalinos, Vassilis Voudris, Kyriakoula Merakou, Kallirrhoe Kourea and Anastasia Barbouni

Survey Questionnaire

The questions and correct answers used to calculate the knowledge score are marked with red color letters.

1. Age

2. Gender

- Woman
- Male

3. Occupation

- Nurse
- General practitioner
- Cardiologist
- Dentist
- Internist
- Pediatrician
- Respiratory physician

4. Working sector

- Public sector
- Private sector

5. How long (years) have you been working as a nurse or a physician?

6. Please report your perceived level of knowledge about smoking.

- Very high
- Fairly high
- Moderate
- Low

7. How often do you see patients who smoke in your working environment?

- Daily
- Less than daily, but at least 3 days per week
- 1–2 days per week
- Less than 1 day per week

8. Do you currently smoke?

- Yes
- No

8a. (only for those who are current smokers)

Do you seriously consider quitting smoking in the near future?
- Yes
- No

9. Have you ever smoked in the past?

- Yes
- No

9a (only for those who have smoked in the past)

How did you quit smoking?

- By using oral medications
- By using nicotine replacement therapies (patch, chewing gum, spray, *etc.*)
- By using systematic psychological support
- By using other methods (acupuncture, hypnotherapy, *etc.*)
- Without using any aid

10. Based on your opinion/knowledge, please score the health risk of each of the following products from 1 (lowest risk) to 10 (highest risk).

- Tobacco cigarettes
- Snus
- Electronic cigarettes
- Nicotine replacement therapies
- Oral smoking cessation medications (varenicline, bupropion)

11. Based on your opinion/knowledge, please score the health risk of each of the following components of tobacco cigarettes from 1 (lowest risk) to 10 (highest risk).

- Nicotine
- inhaled smoke
- Carbon monoxide
- Tar
- Tobacco

12. Based on your opinion/knowledge, what is the contribution of nicotine to the development of smoking-related diseases?

- Extremely important
- Very important
- Important
- Less important
- Minimal

13. Based on your opinion/knowledge, what is the contribution of nicotine to the development of smoking-related lung cancer?

- Extremely important
- Very important
- Important
- Less important
- Minimal

14. Based on your opinion/knowledge, what is the contribution of nicotine to the development of smoking-related cancer in other organs (besides the lungs, e.g., urinary bladder, pancreas, gastrointestinal tract, *etc.*)?

- Extremely important
- Very important
- Important
- Less important
- Minimal

15. Based on your opinion/knowledge, what is the contribution of nicotine to the development of smoking-related atherosclerosis in coronary and peripheral arteries?

- Extremely important
- Very important
- Important
- Less important
- Minimal

16. Compared to smoking, you consider the health risk of nicotine replacement therapies:

- Higher
- Equal
- Lower
- Do not know

17. Compared to smoking, you consider the dependence potential of nicotine replacement therapies:

- Higher
- Equal
- Lower
- Do not know

18. What is the approximate success rate of nicotine replacement therapies in smoking cessation according to the literature?

- Over 50% at 1 year follow-up
- 30%–50% at 1 year follow-up
- 10%–30% at 1 year follow-up
- Less than 10% at 1 year follow-up

19. Nicotine in nicotine replacement therapies is:

- Extracted from tobacco
- Synthetically produced

20. Do you believe that long-term (>6 months) use of nicotine replacement therapies to reduce or quit smoking is relatively safe?

- Yes
- No

21. Would you recommend the long-term (>6 months) use of nicotine replacement therapies for those who cannot reduce or quit smoking with short-term use?

- Yes
- No

22. Compared to smoking, you consider the health risk of electronic cigarettes:

- Higher
- Equal
- Lower
- Do not know

23. Compared to smoking, you consider the dependence potential of electronic cigarettes:

- Higher
- Equal
- Lower
- Do not know

24. Nicotine in electronic cigarettes is:

- Extracted from tobacco
- Synthetically produced

25. Would you recommend electronic cigarettes as a substitute for smoking to smokers who refuse to take medications for smoking cessation?

- Yes
- No

26. Would you recommend electronic cigarettes to smokers who failed to quit with other methods?

- Yes
- No

27. Which of the following are correct concerning electronic cigarette?

- They contain tobacco
- There is combustion
- E-liquid ingredients are approved for inhalation
- Working temperature in electronic cigarettes is lower than in tobacco cigarettes
- They have official quality certificates
- There are electronic cigarettes without nicotine

28. Do you consider e-cigarettes effective in substituting smoking?

- Yes
- No

29. What do you think regulation on electronic cigarettes should include? (You can select more than one answer)

- To be available only through prescription
- To ban nicotine
- To ban flavors
- To ban sales to youngsters (<18 years)
- To prohibit the use in public places
- To be licensed as medicinal products
- To be sold only in pharmacies
- To reduce the variability of products on the market
- To ban advertising or promoting them as substitutes for smoking
- To include a warning that they are equally harmful to smoking

30. Do you know the European Union regulatory framework on electronic cigarettes?

- Yes
- No

© 2016 by the authors; licensee MDPI, Basel, Switzerland. This article is an open access article distributed under the terms and conditions of the Creative Commons by Attribution (CC-BY) license (http://creativecommons.org/licenses/by/4.0/).
